# Economic Burdens of Type 2 Diabetes Hospital Visits with Hypoglycemic Episodes in the Tertiary Care Setting in Thailand

**DOI:** 10.3390/healthcare11070949

**Published:** 2023-03-24

**Authors:** Natapong Kosachunhanun, Danil Wongsa, Unchalee Permsuwan

**Affiliations:** 1 Department of Internal Medicine, Faculty of Medicine, Chiang Mai University, Chiang Mai 50200, Thailand; natapong.k@cmu.ac.th (N.K.);; 2 Center for Medical and Health Technology Assessment (CM-HTA), Department of Pharmaceutical Care, Faculty of Pharmacy, Chiang Mai University, Chiang Mai 50200, Thailand; 3 Department of Pharmaceutical Care, Faculty of Pharmacy, Chiang Mai University, Chiang Mai 50200, Thailand

**Keywords:** hypoglycemia, type 2 diabetes, economic burdens, costs

## Abstract

This study aimed to estimate the economic burden of hypoglycemia among people with type 2 diabetes (T2D) treated in a tertiary care setting. An electronic database of the largest university-affiliated hospital in northern Thailand was retrieved from 2015 to 2020 using the International Classification of Diseases 10th Revision (ICD-10) code E10.xx–E14.xx, or for patients receiving diabetes treatment at least twice for a 6-month period. All records were screened for hypoglycemia using an ICD-10 code E16.0–E16.2 or for having blood glucose <70 mg/dL. All costs related to outpatient visits or inpatient admissions were recorded. During the study period, T2D visits totaled 861,969. The annual incidence rate of hypoglycemia was 2.3 per 1000 visits, while the admission rate was 3.9 per 10,000 visits. The mean length of stay was 4.5 ± 10.1 days. The costs of hypoglycemia were USD 831.1 per admission and USD 182.2 per outpatient visit. The important cost driver for outpatients was drugs (USD 137.1), while for inpatients, this constituted services (USD 299.9). Hypoglycemia poses a substantial financial burden and increases the use of healthcare resources. Selecting the most cost-effective treatments with clinical evidence of the lower risk of hypoglycemia, especially newer insulin preparations, will provide the greatest likelihood of improving clinical outcomes and reducing the economic burden.

## 1. Introduction

An estimated 10.5% of the global population aged 20 to 79 years old are currently coping with diabetes [1]. The prevalence of diabetes among Thais aged over 20 years old is 9.9% (10.8% among women and 8.9% among men) [1]. Without proper treatment, the disease will progress to diabetes-related complications, affecting the quality of life and the economic burdens of patients and society. Moreover, hypoglycemia was found in 10.5% of elderly people with type 2 diabetes in the previous 12 months from a retrospective medical chart review study conducted in a tertiary diabetes center in Thailand [2]. Hypoglycemia related to diabetic management (blood sugar level below 70 mg/dL) is a fact of life among people with diabetes, which causes physical and psychological morbidity. Moreover, a 2-fold increase in the risk of cardiovascular morbidity was observed amongst patients with type 2 diabetes and severe hypoglycemia [3,4]. Additionally, hypoglycemia can create a major barrier to achieving better glycemic control by therapeutic inertia due to patients’ and physicians’ fears of hypoglycemia [5]. Hypoglycemic events may negatively impact health-related quality of life and life expectancy and use more healthcare resources than those without hypoglycemic events [6,7]. A US costing study determined the total financial burden of hypoglycemia to be USD 1.84 billion in 2009 [8]. In Canada, an average cost of USD 7000 for a 7-day admission was due to hypoglycemia [9], while in Scotland, the daily cost was USD 303 per person [10]. In Asia, a Korean study in secondary and tertiary hospitals estimated that medical costs for a hypoglycemic event ranged from USD 17.28 to 1857, respectively [11]. In Malaysia, the length of a hospital stay for severe hypoglycemia was 2 to 26 days, with a cost of USD 2289 [12]. A recent study in Taiwan showed that a hypoglycemic event among people with type 2 diabetes revealed an additional USD 1353 on average annual direct healthcare costs with an additional 5.9 days of hospitalization, compared with people with type 2 diabetes without a hypoglycemic event [13]. Existing studies reported the cost of severe hypoglycemia in Thailand to be around THB 10,574 to 28,494 per event [14,15,16] or about 288.91 to 778.52 USD (with an exchange rate of THB/USD 36.6). These costs might be outdated, since there have been changes in antidiabetic drugs over the years. In addition, the costs from previous studies have not focused on longitudinal follow-up diabetes treatment. The updated cost data of hypoglycemic events are needed. Therefore, this study was conducted to fill the gap by estimating the healthcare resources and the economic burden of hypoglycemia treatment among people with type 2 diabetes visiting a tertiary medical care setting in Thailand.

## 2. Materials and Methods

### 2.1. Study Design and Population

**Figure 1:**
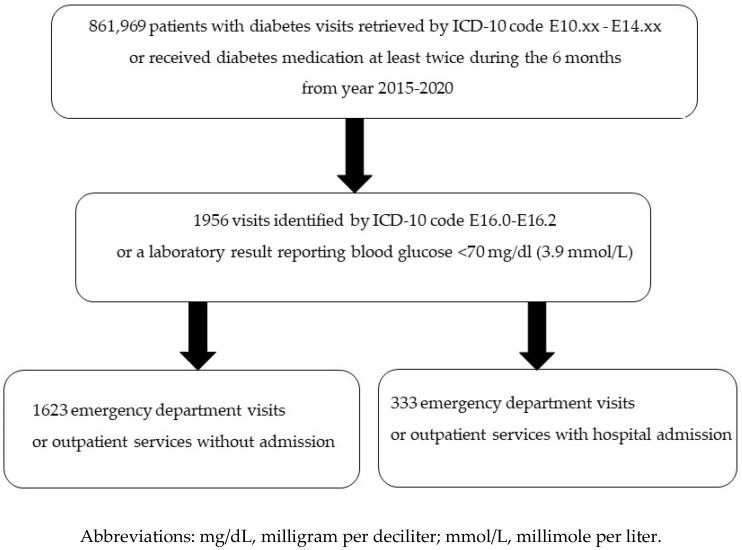
Patient selection flow chart.

The study was approved by the Institutional Review Board of the Faculty of Medicine of Chiang Mai University (MED-2564-08015), and patient consent to review their medical records was not required owing to the database study. The electronic database from Maharaj Nakorn Chiang Mai Hospital, which is the largest university-affiliated tertiary care hospital in northern Thailand, was retrieved for 6 years. Patient records were retrieved using the International Classification of Diseases 10th Revision (ICD-10) code E10.xx–E14.xx for patients receiving diabetes treatment at least twice for a 6-month period. Then, we screened all patient records that indicated severe hypoglycemic events using the ICD-10 code E16.0–E16.2 as either a primary or secondary diagnosis or that had a laboratory result with blood glucose <70 milligrams per deciliter (mg/dL) or 3.9 millimoles per liter (mmol/L). The patient records were finally separated as outpatient visits and admissions to the hospital, as shown in Figure 1. Once patients with type 2 diabetes records had been selected, we retrieved the database of healthcare resources in terms of the number of outpatient visits, the number of hospital admissions, the length of stay (LOS) and the costs of hypoglycemic events from outpatient visits and inpatient admissions.

This study was approved by the research ethics committee, Faculty of Medicine, Chiang Mai University, Thailand (study code: MED-2564-08015).

### 2.2. Data Analysis

Healthcare resource use and costs were calculated from the perspective of the healthcare provider. Hence, the analyses accounted for only direct medical costs, which were composed of drugs, laboratory tests, chest X-rays, surgeries and operations, services, instruments and food and room. All costs and healthcare resource-use items were assessed for all the included patient visits from 2015 to 2020, investigating total costs and average costs ± the standard deviation (SD). In addition, the costs of hypoglycemic events were disaggregated into individual cost items such as medications, laboratory tests, chest X-rays, surgeries or operations, instruments and food and room services. A multivariate generalized linear model (GLM) with a gamma distribution and a log link was used due to the skewness of our cost data. All costs were inflated using the consumer price index in the medical care category of 2021 and reported in Thai baht (THB) and US dollars (USD). All costs were converted to USD at the rate of THB/USD 36.6 as of 14 September 2022 [17].

## 3. Results

Over a 6-year period, there were 861,969 diabetic patient visits. Of those, 1956 visits were associated with hypoglycemic events with blood glucose levels <70 mg/dL. This resulted in an annual incidence rate of hypoglycemic visits amounting to 2.3 per 1000 visits (Table 1). Of those, 1623 visits comprised 893 patients attending the emergency department or receiving outpatient services without hospital admissions, while 333 visits were from 254 unique patients attending the emergency department or receiving outpatient services and were eventually admitted to the hospital owing to severe hypoglycemia. The average age ± standard deviation (SD) for those admitted to the hospital was 66.2 ± 13.8 years. Of all the 254 admitted patients, 53.9% were female. The trend of hospital admissions from severe hypoglycemic events increased from 13.5 to 32.4% from 2015 to 2020. This yielded a total of 17% admissions, and the hospital admission rate was 3.9 per 10,000 visits yearly (Table 1). The mean LOS ± SD was 4.5 ± 10.1 days, while the median LOS was 2 days with an interquartile range of 1 to 4 days.

Of all 333 hospital admissions, the majority of patients received medication reconciliation with previously prescribed antidiabetic treatment. Patients mostly received two anti-diabetic drugs (39.0%), followed by one antidiabetic drug (36.9%). Insulin accounted for 61.3%, followed by biguanides (38.1%) and sulfonylureas (35.7%). Among insulin users, 194 (58.3%) used only one insulin preparation, while the remaining 10 (3.0%) used two insulin preparations. The major insulin preparation was premixed insulin (52.3%). All details are shown in Table 2.

The total cost of all outpatient visits with hypoglycemic episodes ranged from THB 1.3 to 3.0 million (USD 36,374 to 82,975). The total cost was disaggregated into the cost of drugs, laboratory work, chest X-rays and others. Drugs contributed the highest proportion to the total cost, followed by laboratory costs. The average cost for the 6-year period was THB 2,174,224 (USD 59,405). For inpatient admissions, the total cost ranged from THB 0.7 to 3.2 million (USD 18,069 to 88,949). Of all the annual costs, the top three cost items were inpatient services, laboratory work and drugs. The average cost for the 6-year period was THB 1,688,216 (USD 46,126). All details are shown in Table 3.

**Figure 2:**
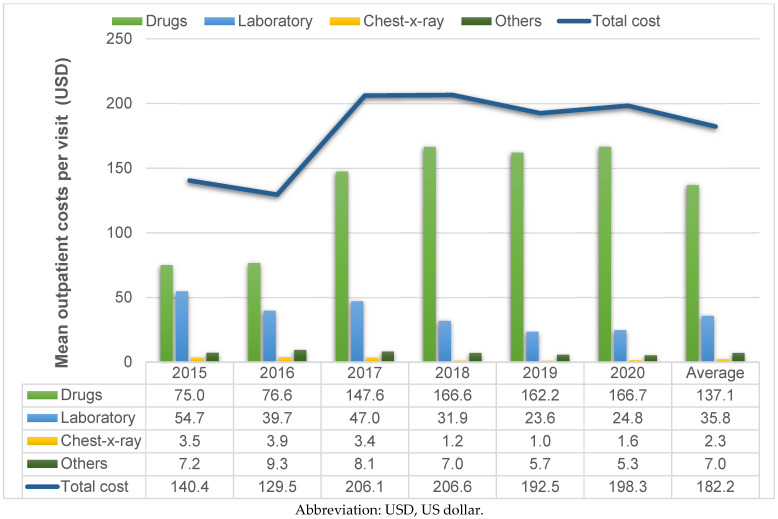
Mean outpatient costs per visit over a 6-year period.

**Figure 3:**
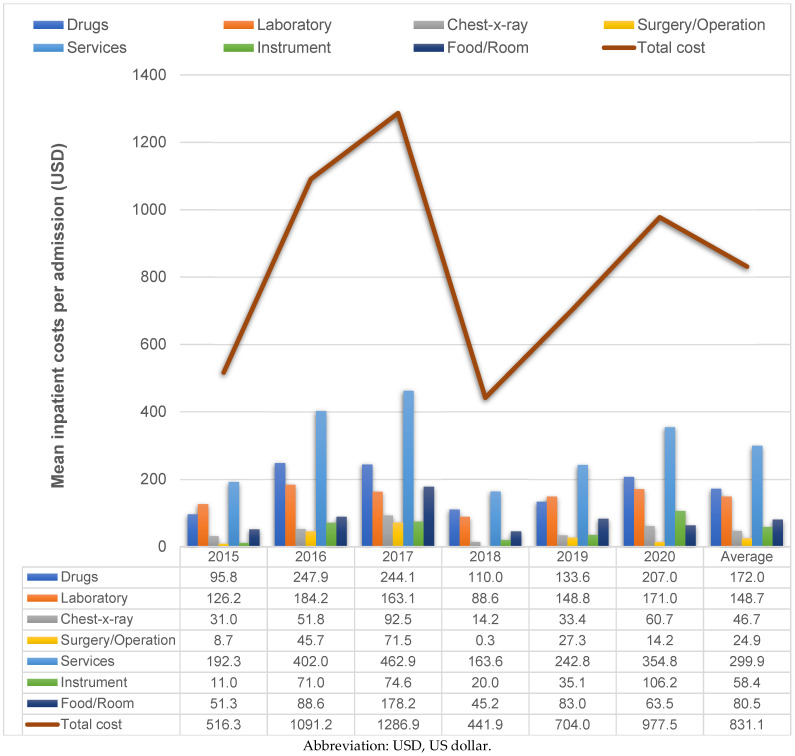
Mean inpatient costs per admission over a 6-year period.

The mean cost per outpatient visit ranged from USD 129.5 to USD 206.6 over 6 years, with an average cost ± SD of USD 182.2 ± 464.6 per outpatient visit (Figure 2). The mean cost per admission ranged from USD 516.3 to USD 1286.9 for the 6-year period. The average cost ± SD was USD 831.1 ± 3038.9 per admission (Figure 3). All details of the average cost ± SD in each cost item are shown in Table 4.

According to a multivariable GLM, the length of stay had a significant impact on the total cost of inpatient visits. It was found that 1 additional day of hospitalization incurred an additional cost of THB 1945 (USD 53.1), as shown in Table 5.

## 4. Discussion

Hypoglycemia is often one of the safety outcomes of diabetes therapy. The high frequency of hypoglycemic events that occur results in a poor quality of life and a substantial personal and societal economic burden [6,7]. Thus, its costs are usually considered in the economic evaluation and budget impact analysis of antidiabetic drugs for type 2 diabetes treatment. This study aimed to estimate the healthcare resources and the financial burden of hospital visits from hypoglycemic episodes among people with type 2 diabetes from both outpatient and inpatient settings. The incidence rate of hypoglycemia in this study was 2.3 per 1000 visits yearly; the admission rate was 3.9 per 10,000 visits yearly, with two-thirds of patients using insulin and almost exclusively using premixed insulin preparations. Our findings might be slightly higher than those of other observational studies reporting hospitalizations or emergency department visits for hypoglycemia of 0.2 (patients treated without insulin or sulfonylurea) to 2.0 (insulin or sulfonylurea users) per 100 person-years [18]. However, a randomized controlled trial reported various rates of severe hypoglycemia ranging from 0.7 to 12 per 100 person-years [18]. Comparing the reported rates of hypoglycemia can be difficult because a marked heterogeneity exists regarding how hypoglycemia is defined, measured and reported in each study.

Although the costs of diabetes care have been estimated in several studies in Thailand, estimating diabetes care costs varies depending on hospital care levels, the presence of diabetes complications and so on. This study reported both the costs of inpatient admission and outpatient costs, which several cost-effectiveness studies in Thailand ignored [16,19]. This might be owing to a limitation of available published data on outpatient costs for people with type 2 diabetes and hypoglycemia. This study ensured that outpatient visits were related to hypoglycemia through the laboratory results on the dates patients visited the hospital. We found that the number of visits with blood glucose <70 mg/dL totaled 1956 during the 6-year period or about 326 visits yearly. Of all such visits, 1623 (83%) comprised outpatient or emergency visits only. This incurred the average outpatient cost of THB 6669 per visit (USD 182.2). Drug costs contributed about 72% of the average total cost.

For hospital admissions due to hypoglycemia, the average LOS ± SD was 4.5 ± 10.1 days in this study, which was close to the LOS of 4.3 days for patients with type 2 diabetes in Malaysia [20]. However, our findings indicated shorter lengths of admission compared with those in India (8 to 10 days), China (8.9 to 15.4 days) and another tertiary-care medical school hospital in Bangkok (8.5 days) [20]. This might be due to the higher percentage of patients with type 2 diabetes with any complications admitted in the study by Goldhaber-Fiebert JD et al. [20]. Another study using a nested case–control design in Taiwan concluded that cases or patients with hypoglycemic events experienced a substantially higher economic burden than the matched cohort or patients without hypoglycemic events [13]. Patients with type 2 diabetes and a hypoglycemic event incurred an additional USD 1353 on average in annual direct healthcare costs and an additional 5.9 days in hospital compared with the matched cohort [13]. In this study, it was found that 1 additional day of hospitalization incurred an extra THB 1945 (USD 53.1). The treatment costs of a hypoglycemic event incurred in Thailand might be lower than the costs incurred in Taiwan. This might be due to the differences in healthcare systems, reimbursement policies, the acquisition of drug costs and so on.

As expected, the total cost of diabetes treatment was substantially higher for hospital admissions than outpatient visits. In this study, the average total cost of hospital admissions was about 4.6 times greater than that of outpatient visits (THB 30,418 vs. THB 6669 or USD 831.1 vs. USD 182.2). The outpatient and inpatient costs incurred in this study accounted for only direct medical costs. Drug costs were the most influential contributors to outpatient costs and were the second-highest contributors to inpatient costs. Our findings show that the cost of inpatient admissions at the tertiary-care level was much higher than the inpatient costs reported (USD 199.75 in 2008) from a district hospital located in northeast Thailand. One explanation is the healthcare system in Thailand. Patients with diabetes and controlled blood glucose levels usually receive routine monitoring check-ups at a primary care unit close to their homes. Once their clinical symptoms worsen, they are referred to higher healthcare levels, where human resources and healthcare facilities are more advanced.

With the study design of retrospective database analyses, several limitations were encountered. Firstly, we did not collect direct nonmedical costs or indirect costs as a result of visiting the hospital or for having the disease itself. Based on a related diabetes cost study [21], direct nonmedical costs and indirect costs contributed 40 and 37% of the total costs of diabetes treatment, respectively. A complete picture of the economic burden of diabetes should consider the social perspective, including all direct medical costs, direct nonmedical costs and indirect costs. Because this study focused mainly on the healthcare system’s perspective, only direct medical costs were included. Secondly, the time horizon in this study was over a 6-year period, so changes in the treatment guidelines may have occurred, such as using minimized hypoglycemia as the management need for treatment choice [22]. The drug cost contribution for outpatient visits indicated an upward trend from 53 to 84% during the 6-year period. This may be attributable to the selection of newer drug generations with a lower risk of hypoglycemia, albeit higher costs, including dipeptidyl peptidase-4 inhibitors, sodium-glucose cotransporter-2 inhibitors and glucagon-like peptide-1 agonists.

Although this study possessed these limitations, its findings could be used as important inputs for further studies, such as in the economic evaluation or budget impact analysis of antidiabetic drugs or for other health technologies related to hypoglycemia. This is because it reflects the real-world treatment costs of hypoglycemia among people with diabetes as opposed to protocol-driven costs based on restrictive patient inclusion/exclusion criteria, which are often used in randomized controlled trials. Another issue is related to the generalizability of the findings of this study. Due to the different treatment contexts of type 2 diabetes among different countries, the financial burden associated with hypoglycemic episodes could vary.

## 5. Conclusions

Hypoglycemic hospital visits pose a substantial financial burden and increase the use of healthcare resources in type 2 diabetes treatment. Selecting the most cost-effective treatments with clinical evidence of lower hypoglycemia risk, especially newer insulin preparations, will have the greatest likelihood of improving clinical outcomes and reducing the economic burden.

## Figures and Tables

**Table 1:** Healthcare resource use and incidence rate.

	Year						Total
	2015	2016	2017	2018	2019	2020	
Total visits with type 2 diabetes	93,860	99,363	106,506	175,925	189,385	196,930	861,969
Number of visits with blood glucose <70 mg/dL	259	285	337	363	431	281	1956
Incidence rate of visits with hypoglycemia ^1^ (per 1000 visits per year)	2.8	2.9	3.2	2.1	2.3	1.4	2.3
Number of inpatients	32	34	35	46	66	72	285 ^2^
Number of admissions	35	37	40	52	78	91	333
Admissions ^3^ (percent)	13.5	13.0	11.9	14.3	18.1	32.4	17.0
Admission rate ^4^ (per 10,000 visits per year)	3.7	3.7	3.8	3.0	4.1	4.6	3.9

^1^ Incidence rate = (number of visits with blood glucose <70 mg/dL × 1000)/total visits with type 2 diabetes. ^2^ Some patients readmitted in the same year or different years; all unique hospital numbers totaled 254 patients. ^3^ Admissions = (number of admissions × 100)/number of visits with blood glucose <70 mg/dL. ^4^ Admission rate = (number of admissions × 10,000)/total visits with type 2 diabetes.

**Table 2:** Antidiabetic treatment used among admitted patients.

	Total of 333 Admissions (%)	
Number of antidiabetic drugs patients received		
5	1	(0.3)
4	8	(2.4)
3	69	(20.7)
2	130	(39.0)
1	123	(36.9)
Diet control	2	(0.6)
Classes of antidiabetic drugs		
Insulin -1 insulin preparation -2 insulin preparations	204 194 10	(61.3) (58.3) (3.0)
Biguanides	127	(38.1)
Sulfonylureas	119	(35.7)
Thiazolidinedione	81	(24.3)
Dipeptidyl peptidase inhibitors	60	(18.0)
Sodium glucose cotransporter inhibitors	4	(1.2)
Glucagon-like peptide-1 receptor agonists	1	(0.3)
Combined drugs	17	(5.1)
Other classes	4	(1.2)
Insulin		
First insulin preparation	204	(61.3)
-Premixed insulin	174	(52.3)
-Long-acting insulin	20	(6.0)
-Intermediate-acting insulin	10	(3.0)
Second insulin preparation	10	(3.0)
-Rapid-acting insulin	6	(1.8)
-Intermediate-acting insulin	3	(0.9)
-Premixed insulin	1	(0.3)

**Table 3:** Total and disaggregated cost items of outpatients and inpatient visits.

Cost Item	Year 2015		Year 2016		Year 2017		Year 2018		Year 2019		Year 2020	
	Value	%	Value	%	Value	%	Value	%	Value	%	Value	%
Cost of outpatient visit THB (USD)												
Drugs	711,019	53	798,829	59	1,820,045	72	2,213,188	81	2,558,889	84	1,714,570	84
	(19,426.7)		(21,825.9)		(49,728.0)		(60,469.6)		(69,915.0)		(46,846.2)	
Laboratory work	518,897	39	414,204	31	579,777	23	423,183	15	371,504	12	254,837	12
	(14,177.5)		(11,317.0)		(15,840.9)		(11,562.4)		(10,150.4)		(6962.8)	
Chest X-ray	33,457	3	40,424	3	42,020	2	15,51	1	15,996	1	16,059	1
	(914.1)		(1104.5)		(1148.1)		(427.6)		(437.0)		(438.8)	
Others	67,942	5	97,357	7	100,166	4	93,131	3	89,856	3	54,345	3
	(1856.3)		(2660.0)		(2736.8)		(2544.6)		(2455.1)		(1484.8)	
Total cost	1,331,314	100	1,350,813	100	2,542,008	100	2,745,153	100	3,036,245	100	2,039,811	100
	(36,374.7)		(36,907.5)		(69,453.8)		(75,004.2)		(82,957.5)		(55,732.5)	
Average	2,174,224 (59,405.03)											
Cost of inpatient visit THB (USD)												
Drugs	122,709	19	335,696	23	357,327	19	209,412	25	381,314	19	689,552	21
	(3352.7)		(9172.0)		(9763.0)		(5721.6)		(10,418.4)		(18,840.2)	
Laboratory work	161,620	24	249,388	17	238,712	13	168,576	20	424,729	21	569,549	17
	(4415.9)		(6813.9)		(6522.2)		(4605.9)		(11,604.6)		(15,561.4)	
Chest X-ray	39,732	6	70,149	5	135,383	7	27,069	3	95,238	5	202,207	6
	(1085.6)		(1916.6)		(3699.0)		(739.6)		(2602.1)		(5524.8)	
Surgeries/	11,132	2	61,948	4	104,717	6	570	0.1	78,067	4	47,276	1
operations	(304.1)		(1692.6)		(2861.1)		(15.6)		(2133.0)		(1291.7)	
Services	246,341	37	544,423	37	677,734	36	311,282	37	693,182	34	1,181,732	36
	(6730.6)		(14,874.9)		(18,517.3)		(8505.0)		(18,939.4)		(32,287.8)	
Instruments	14,061	2	96,166	7	109,252	6	38,041	5	100,122	5	353,712	11
	(384.2)		(2627.5)		(2985.0)		(1039.4)		(2735.6)		(9664.2)	
Food/room	65,736	10	119,951	8	260,846	14	86,031	10	237,090	12	211,525	6
	(1796.1)		(3277.3)		(7126.9)		(2350.6)		(6477.9)		(5779.4)	
Total cost	661,331	100	1,477,719	100	1,883,971	100	840,980	100	2,009,741	100	3,255,553	100
	(18,069.1)		(40,374.8)		(51,474.6)		(22,977.6)		(54,911.0)		(88,949.5)	
Average	1,688,216 (46,126.11)											

Abbreviations: THB, Thai Baht; USD, US dollar.

**Table 4:** Average costs of outpatient and inpatient visits.

Costs	Year 2015		Year 2016		Year 2017		Year 2018		Year 2019		Year 2020	
	Mean	SD	Mean	SD	Mean	SD	Mean	SD	Mean	SD	Mean	SD
Average outpatient cost per visit THB (USD)												
Drugs	2745 (75.0)	8397 (229.4)	2803 (76.6)	9504 (259.7)	5401 (147.6)	15,138 (413.6)	6097 (166.6)	17,307 (472.9)	5937 (162.2)	20,546 (561.4)	6102 (166.7)	20,859 (569.9)
Laboratory Work	2003 (54.7)	5847 (159.8)	1453 (39.7)	3360 (91.8)	1720 (47.0)	5257 (143.6)	1166 (31.9)	2317 (63.3)	862 (23.6)	887 (24.2)	907 (24.8)	997 (27.2)
Chest X-ray	129 (3.5)	1075 (29.4)	142 (3.9)	970 (26.5)	125 (3.4)	671 (18.3)	43 (1.2)	222 (6.1)	37 (1.0)	188 (5.1)	57 (1.6)	364 (9.9)
Others	262 (7.2)	606 (16.6)	342 (9.3)	925 (25.3)	297 (8.1)	927 (25.3)	257 (7.0)	649 (17.7)	208 (5.7)	529 (14.4)	193 (5.3)	317 (8.7)
Total cost	5140 (140.4)	9997 (273.1)	4740 (129.5)	10,127 (276.7)	7543 (206.1)	16,19 (442.5)	7562 (206.6)	17,684 (483.2)	7045 (192.5)	20,667 (564.7)	7259 (198.3)	20,964 (572.8)
Average outpatient cost per visit ± SD 6669 ± 17,005 (182.2 ± 464.6)												
Average inpatient cost per admission THB (USD)												
Drugs	3506 (95.8)	6079 (166.1)	9073 (247.9)	21,956 (599.9)	8933 (244.1)	31,385 (857.5)	4027 (110.0)	13,394 (366.0)	4889 (133.6)	10,954 (299.3)	7578 (207.0)	50,300 (1374.3)
Laboratory Work	4618 (126.2)	5752 (157.2)	6740 (184.2)	8979 (245.3)	5968 (163.1)	14,214 (388.4)	3242 (88.6)	5505 (150.4)	5445 (148.8)	11,182 (305.5)	6259 (171.0)	20,874 (570.3)
Chest X-ray	1135 (31.0)	2472 (67.5)	1896 (51.8)	4241 (115.9)	3385 (92.5)	11,513 (314.6)	521 (14.2)	1277 (34.9)	1221 (33.4)	3535 (96.6)	2222 (60.7)	9294 (253.9)
Surgeries/ operations	318 (8.7)	872 (23.8)	1674 (45.7)	7441 (203.3)	2618 (71.5)	14,286 (390.3)	11 (0.3)	79 (2.2)	1001 (27.3)	3942 (107.7)	520 (14.2)	3286 (89.8)
Services	7038 (192.3)	9757 (266.6)	14,714 (402.0)	23,151 (632.5)	16,943 (462.9)	57,129 (1560.9)	5986 (163.6)	16,243 (443.8)	8887 (242.8)	25,302 (691.3)	12,986 (354.8)	57,756 (1578.0)
Instruments	402 (11.0)	645 (17.6)	2599 (71.0)	4980 (136.1)	2731 (74.6)	10,042 (274.4)	732 (20.0)	2202 (60.2)	1284 (35.1)	2878 (78.6)	3887 (106.2)	27,781 (759.0)
Food/room	1878 (51.3)	3232 (88.3)	3242 (88.6)	5948 (162.5)	6521 (178.2)	28,608 (781.6)	1654 (45.2)	3848 (105.1)	3040 (83.0)	7524 (205.6)	2324 (63.5)	8706 (237.9)
Total cost	18,895 (516.3)	25,413 (694.4)	39,938 (1091.2)	63,738 (17,415)	47,099 (1286.9)	153,035 (4181.3)	16,173 (441.9)	40,907 (1117.7)	25,766 (704.0)	54,365 (1485.4)	35,775 (977.5)	172,517 (4713.6)
Average inpatient costs per admission ± SD 30,418 ± 111,224 (831.1 ± 3038.9)												

Abbreviations: SD, standard deviation; THB, Thai Baht; USD, US dollar.

**Table 5:** Baseline predictors of the total cost of inpatient visits in a multivariate model.

Variables	Coefficient	Standard Error	*p*-Value
Intercept	9.3337	0.5514	<0.001
Age	−0.0033	0.0048	0.489
Female	−0.0278	0.1442	0.847
Length of stay	0.1586	0.0319	<0.001
Insulin use	−0.2285	0.2074	0.271
Metformin use	−0.2126	0.1657	0.199
Sulfonylurea use	0.0702	0.2088	0.737
Thiazolidinedione use	−0.1109	0.1387	0.424
DPP-4 inhibitor use	−0.2449	0.1914	0.201
GLP-1 agonist use	−0.3255	0.1833	0.076
SGLT-2 inhibitor use	−0.0240	0.2833	0.933

Equation: ln(total cost) = 9.3337 + 0.1586 x length of stay. Length of stay = 0. ln(total cost) = 9.3337. Total cost = exp(9.3337) = 11,312 THB. Length of stay = 1. ln(total cost) = 9.3337 + 0.1586 x 1 = 9.4923. Total cost = exp(9.4923) = 13,257 THB. Total cost for 1 additional hospitalization = 13,257 − 11,312 = 1945 THB. Abbreviations: DPP-4, dipeptidyl peptidase-4; GLP-1, glucagon-like peptide-1; SGLT-2, sodium-glucose cotransporter-2.

## Data Availability

Not applicable.
